# After-Dinner Speeches of Americans

**Published:** 1883-09

**Authors:** 


### AFTER-DINNER SPEECHES OF AMERICANS.

It has often been debated whether it is to climate, to race, to the influence of free institutions, or to some other subtle cause, that men owe the gift of eloquence, and in a little book called "Daniel Webster and his Contemporaries" it is stated that the prince of American orators was firm in his conviction that, as a predisposing cause, climate outweighed all the rest. If such be the case, the climate of these islands is evidently less calculated to create good speakers than that of the North American Continent, as among us there is not one man fluent of tongue against ten to whom our kinsfolk in the United States can point. Take, for instance, that rarest and, to Englishmen, most difficult accomplishment of after-dinner oratory, and who that has attended a public dinner in London and another in New York can doubt upon which side the Atlantic the superiority in this respect lies? About a quarter of a century since Richard Cobden, then at his apogee as one of the most persuasive and practical speakers in the House of Commons, chanced to pay a visit to Chicago, in order to look after an investment he had made in the shares of the Illinois Central Railroad. There a complimentary dinner was given to him, at which some forty guests were included. Among them was a fairly representative sprinkling of the bankers, merchants, lawyers, speculators, and what not of that wonderful young city; and, dinner being over, speaking was freely indulged in, the verdict of all present—Englishmen and Americans—being that the worst speeches of the evening came from Richard Cobden's lips. People were wont twenty years since, to regard Charles Dickens as an after-dinner speaker almost without a superior in either hemisphere. The last visit of the great novelist to the United States sufficed, however, to dissipate the illusion under which his countrymen were laboring. "Finally, gentlemen," remarked Charles Dickens, at the banquet given to him in New York on April 18, 1868, "I do not believe that, from every honest mind, upon both sides of the Atlantic, there cannot be absent the conviction that it would be better for this globe to be riven by an earthquake, fired by a comet, overrun by an iceberg and abandoned to the Arctic fox and bear, than that it should present the spectacle of these two great nations, each of which has in its own way done so much for freedom, being ever again arrayed the one against the other."

Compare with the hyperbole of this extravagant, or, to use an Americanism, "hifalutin" elocution, the easy grace and quiet dignity of the speech delivered, with admirable effect, by the American Ambassador, Mr. James Russell Lowell, at the dinner recently given by the Institution of Civil Engineers. There were Englishmen present at that dinner who—again to employ an Americanism—

were well calculated "to hold up their end of the plank." The House of Peers was represented by Lord Derby and Lord Kimberley; the House of Commons by Mr. Bright, Sir Richard Cross and Mr. Childers. No one of ordinary culture who chanced to hear or to read the speeches delivered by the English Cabinet Ministers would—to use a phrase of which Robert Southey was very fond—have "had his mind scratched" by any one of them. Mr. Bright indeed, could not altogether get away from party politics, even upon that confessedly neutral ground. But when Mr. Lowell rose, there was in the accents, the delivery, the voice, the manner, and still more in the words of the speaker, that which irresistibly engaged the fancy and riveted the attention of the hearers.—*From the London Telegraph.*

